# Cyanobacterial circadian regulation enhances bioproduction under subjective nighttime through rewiring of carbon partitioning dynamics, redox balance orchestration, and cell cycle modulation

**DOI:** 10.1186/s12934-025-02665-5

**Published:** 2025-03-08

**Authors:** Ashley Gilliam, Natalie C. Sadler, Xiaolu Li, Marci Garcia, Zachary Johnson, Marija Veličković, Young-Mo Kim, Song Feng, Wei-Jun Qian, Margaret S. Cheung, Pavlo Bohutskyi

**Affiliations:** 1 Biological Sciences Division, Earth and Biological Sciences Directorate, Pacific Northwest National Laboratory, Richland, WA USA; 2 Department of Biological Systems Engineering, Washington State University, Pullman, WA USA; 3 Environmental Molecular Sciences Laboratory, Pacific Northwest National Laboratory, Richland, WA USA; 4 Department of Physics, University of Washington, Seattle, WA USA

## Abstract

**Background:**

The industrial feasibility of photosynthetic bioproduction using cyanobacterial platforms remains challenging due to insufficient yields, particularly due to competition between product formation and cellular carbon demands across different temporal phases of growth. This study investigates how circadian clock regulation impacts carbon partitioning between storage, growth, and product synthesis in *Synechococcus elongatus* PCC 7942, and provides insights that suggest potential strategies for enhanced bioproduction.

**Results:**

After entrainment to light-dark cycles, PCC 7942 cultures transitioned to constant light revealed distinct temporal patterns in sucrose production, exhibiting three-fold higher productivity during subjective night compared to subjective day despite moderate down-regulation of genes from the photosynthetic apparatus. This enhanced productivity coincided with reduced glycogen accumulation and halted cell division at subjective night time, suggesting temporal separation of competing processes. Transcriptome analysis revealed coordinated circadian clock-driven adjustment of the cell cycle and rewiring of energy and carbon metabolism, with over 300 genes showing differential expression across four time points. The subjective night was characterized by altered expression of cell division-related genes and reduced expression of genes involved in glycogen synthesis, while showing upregulation of glycogen degradation pathways, alternative electron flow components, the pentose phosphate pathway, and oxidative decarboxylation of pyruvate. These molecular changes created favorable conditions for product formation through enhanced availability of major sucrose precursors (glucose-1-phosphate and fructose-6-phosphate) and maintained redox balance through multiple mechanisms.

**Conclusions:**

Our analysis of circadian regulatory rewiring of carbon metabolism and redox balancing suggests two potential approaches that could be developed for improving cyanobacterial bioproduction: leveraging natural circadian rhythms for optimizing cultivation conditions and timing of pathway induction, and engineering strains that mimic circadian-driven metabolic shifts through controlled carbon flux redistribution and redox rebalancing. While these strategies remain to be tested, they could theoretically improve the efficiency of photosynthetic bioproduction by enabling better temporal separation between cell growth, carbon storage accumulation, and product synthesis phases.

**Supplementary Information:**

The online version contains supplementary material available at 10.1186/s12934-025-02665-5.

## Introduction

Cyanobacteria comprise a promising engineering platform for production of industrial chemicals, fuels and food. These organisms have been engineered to produce various compounds. Examples include alcohols (ethanol, 1-butanol, isopropanol, and glycerol) [1–4], hydrocarbons and fatty acids (ethylene, isoprene, limonene, bisabolene, undecane, and tridecane) [5–11], aliphatic diols [12–14], ketones and aldehydes [15, 16], polyhydroxyalkanoates [17, 18], various organic acids [18–21] and sugars [22–25], and hydrogen [26]. However, despite these significant advances in developing photosynthetic bioproduction systems, their industrial feasibility remains challenging due to insufficient yields. A key limitation is the competition between product formation and cellular carbon demands, where carbon flux must be balanced between growth, storage compounds, and product synthesis. Understanding and optimizing these carbon partitioning processes requires integrated analysis across multiple levels of cellular organization, from molecular mechanisms to overall production efficiency.

The circadian clock, which coordinates cellular functions over 24-hour cycles, may offer solutions to these carbon partitioning challenges. In *Synechococcus elongatus* PCC 7942, this temporal regulation orchestrates carbon flow through central metabolism, controls glycogen storage pools, and gates cell division [27–31]. Notably, the circadian clock acts as a major modulator of *S. elongatus* growth by controlling the transition between cell elongation and division initiation [30, 32, 33], effectively separating biomass accumulation from cell proliferation. More recent studies have shown that *S. elongatus* integrates circadian signals with external cues [31, 34–36] and other cellular processes, including DNA management [37–39], demonstrating sophisticated coordination of cellular activities in response to environmental conditions.

The molecular basis for this temporal control involves a core oscillator complex of KaiA, KaiB, and KaiC proteins, which interacts with the transcription factor RpaA through the histidine kinases SasA and CikA [40–45]. The RpaA master regulator, together with an array of RpaA-controlled sigma-factors and transcriptional factors coordinates the temporal expression of genes involved in photosynthesis, carbon metabolism, and cell division [40, 43, 46, 47], potentially creating distinct windows that favor either growth and storage or product formation. The fitness benefits of such temporal organization have been demonstrated both in vitro [48] and simulated in silico [49], suggesting that understanding and leveraging these natural rhythms could enhance bioproduction efficiency.

Understanding the impact of circadian regulation on bioproduction through control of glycogen metabolism, biomass synthesis and cell division is particularly relevant, as these cellular processes represent major carbon sinks, with glycogen alone accounting for 30–60% of the total biomass [50–52], and thus directly compete with product formation. However, attempts to enhance product yields through blunt knockout of glycogen synthesis have revealed unexpected complexities. While glycogen synthesis represents a competing carbon sink, its complete elimination can paradoxically decrease carbon flux through heterologous pathways in engineered strains [6, 24, 53–57]. Multiple studies suggest that disruption of glycogen synthesis can lead to significant metabolic imbalances, causing reduction in light capture and carbon fixation, as well as increased sensitivity to environmental fluctuations [58–62]. These findings suggest that rather than direct pathway manipulation, understanding and leveraging natural global circadian oversight of carbon partitioning could provide more effective strategies for enhancing production.

Given the complex interplay between circadian regulation and carbon metabolism, this study aims to elucidate how temporal control of cellular processes impacts carbon partitioning between storage, growth, and product formation in cyanobacteria. Using sucrose production as a model system, we investigate how circadian programming influences the efficiency of photosynthetic bioproduction through coordinated regulation of multiple cellular processes. Understanding these regulatory mechanisms will provide crucial insights for metabolic engineering strategies, potentially enabling the rational design of strains with optimized temporal coordination of cell division, glycogen accumulation, and product synthesis. Such knowledge could ultimately lead to significant improvements in both the efficiency and yield of photosynthetic bioproduction systems through approaches that work with, rather than against, natural cellular rhythms.

## Materials and methods

### Organism and maintenance

*Synechococcus elongatus* PCC 7942 CscB/SPS, metabolically engineered to produce and secrete sucrose, was a generous gift from Daniel Ducat (Michigan State University, East Lansing, MI). This sucrose-exporting strain overexpresses genes coding for sucrose phosphate synthase (*sps*) and sucrose permease (*cscB*) upon induction by isopropyl β-d-1-thiogalactopyranoside (IPTG), as described previously [63–65]. Cultures were maintained at 29 ± 2 °C in 1.2 L Roux culture flasks containing 0.4–0.6 L of BG-11 medium supplemented with 1 g L⁻¹ HEPES (pH 8.4, adjusted with NaOH), 25 mg L⁻¹ chloramphenicol, and 25 mg L⁻¹ kanamycin to maintain *cscB* and *sps*. Cultures were diluted to OD_7__5_₀ 0.2 every 4 days. Mixing was achieved using PFTE Octagon Spinbar Magnetic Stirring Bars (9.5 mm × 25.4 mm) at approximately 200 rpm, and cultures were sparged with nitrogen gas containing 2% CO_2_. Light intensity at the flask surface was maintained at approximately 200 µE m⁻² s⁻¹ using Monios-L LED Full Spectrum Grow Lights (measured with LI-250 A Light Meter LI-COR with LI-190SA Quantum Sensor, LI-COR, Inc). Prior to this study, cultures were adapted to a 14:10 light-dark (LD) cycle (light period 06:00–20:00) for approximately 6 months.

### Sucrose production experiment

Prior to the sucrose production experiment, antibiotics were removed from the media by harvesting cells (centrifugation at 6,000 g for 5 min in 50 mL tubes), followed by resuspension in fresh BG-11 medium without antibiotics. This wash step was repeated once. The washed cells were diluted to OD_7__5_₀ 0.2 in fresh BG-11 and split into two cultures to enable measurements during subjective day and subjective night periods. Expression of the sucrose synthesis pathway (*sps*) and export system (*cscB*) was activated 24 h prior to sampling by adding 1 mM IPTG. At time 0, cultures were adjusted to OD_7__5_₀ 0.1 with BG-11 containing IPTG to ensure even light distribution, then divided into three biological replicates of 0.85 L per flask.

**Fig. 1:**
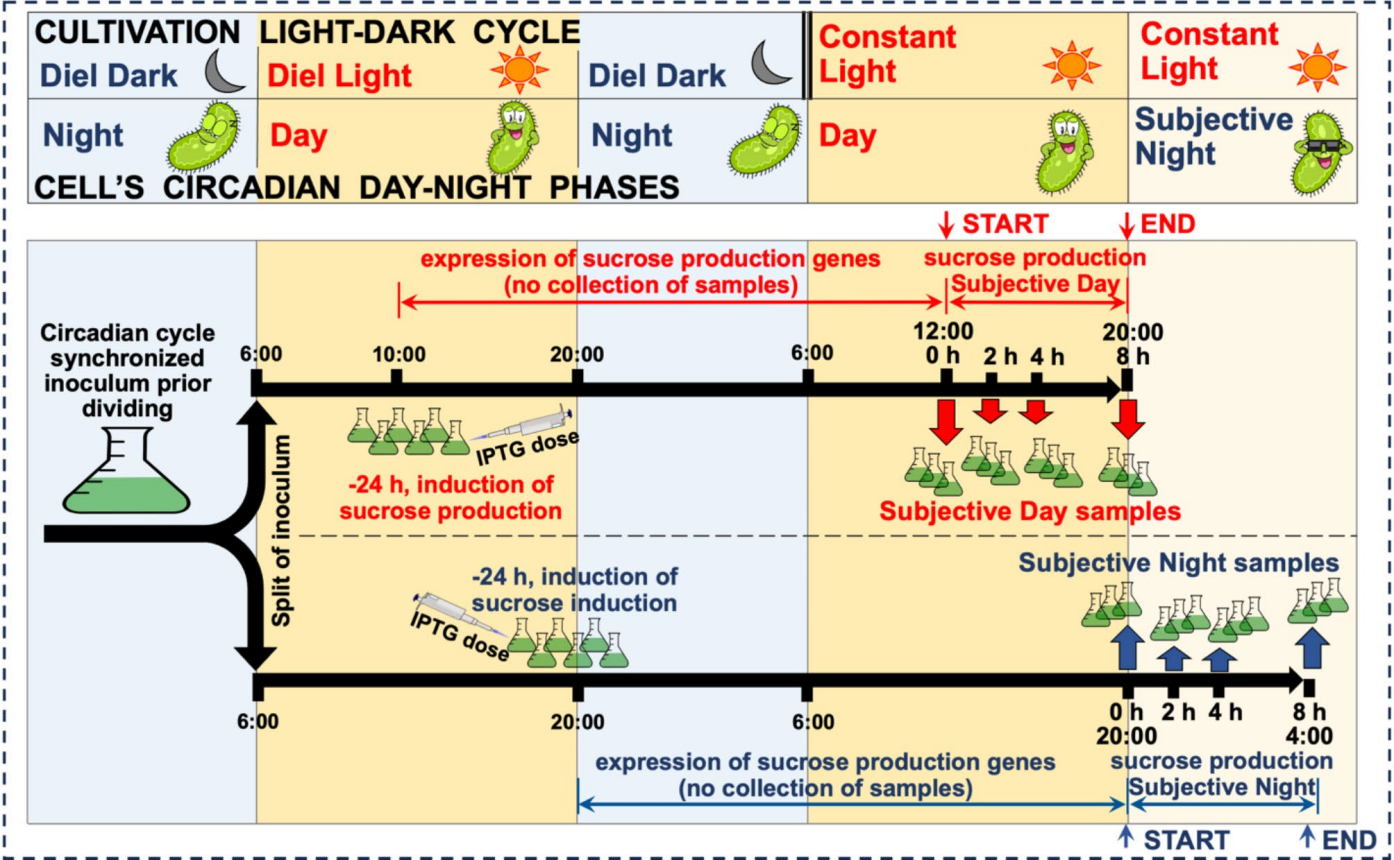
Experimental design and timeline for investigating circadian effects on sucrose production in *S. elongatus* PCC7942 cscB/SPS. The experiment compared subjective day (12:00–20:00) and subjective night (20:00–04:00) conditions under constant light

Culture OD_7__5_₀ was maintained at approximately 0.1 through additions of BG-11 containing IPTG. The pH was maintained above 7.8 (required for efficient *cscB*-based sucrose export [64]) using 1 M NaOH as needed. Both subjective day and night cultures received identical volumes of BG-11 and NaOH to ensure comparable conditions. Time 0 corresponded to 12:00 (noon) for subjective day samples and 20:00 (dusk) for subjective night samples. The experimental design and sampling schedule are detailed in Fig. 1.

### Culture density and cell number quantification

Growth was monitored by measuring optical density at 750 nm (Genesys 20 Spectrophotometer, Thermo Fisher Scientific Inc.) and counting cell numbers (NovoCyte 3000 VYB Flow Cytometer System, Agilent Technologies Inc.). *S. elongatus* cells were identified based on red autofluorescence from photosynthetic pigments and distinct forward scatter (FSC) properties. Samples were analyzed within one hour of collection by diluting 20 µL of culture in 1.98 mL PBS buffer. Cell counting was performed at 50 µL min^− 1^ flow rate to achieve minimum 50,000 *S. elongatus* cell events. Three times of rinses were performed between samples, with one homogenization cycle per sample (1,000 rpm, 10 s). Excitation was performed at 405 nm and 488 nm, with pigment autofluorescence measured using a red filter (660/20 nm). Data analysis was conducted using NovoExpress Software (Agilent Technologies Inc.). Statistical significance of differences in OD750 and cell counts between subjective day and night conditions was assessed using two-tailed Student’s t-tests (*n* = 3 biological replicates). Differences with *p* < 0.05 were considered significant and *p* < 0.01 highly significant.

### Sucrose quantification

Culture samples (8 mL) were collected at 0, 0.5, 1, 2, 4, 6, and 8 h for extracellular sucrose analysis. Cells were removed using 0.2 μm membrane filtration, and filtrates were flash-frozen in liquid N_2_ and stored at -80 °C until analysis. For quantification, 200 µL medium samples were dried under speed vacuum, then dissolved in pyridine and chemically derivatized by methoxyamination and trimethylsilylation as previously described [66]. Serially diluted sucrose standards were similarly prepared and derivatized to generate calibration curves. Derivatized samples and standards were analyzed using an Agilent 7890 gas chromatograph equipped with 5977 mass spectrometry system (Agilent Technologies, USA). The GC oven temperature was initially held at 60 °C, then increased at a rate of 10 °C/min to a final temperature of 325 °C. Data analysis was performed using Agilent MassHunter software (Agilent Technologies, USA), where the peak area of the dominant sucrose fragment (m/z 361) was integrated and quantified against the calibration curves (R² > 0.95). Statistical significance of differences in sucrose levels between subjective day and night conditions was assessed using two-tailed Student’s t-tests (*n* = 3 biological replicates). Differences with *p* < 0.05 were considered significant and *p* < 0.01 highly significant.

### Glycogen quantification

For glycogen analysis, cells from 30 mL culture were collected by centrifugation (6,000 g, 10 min, 4 °C). Cell pellets were flash-frozen in liquid N_2_ and stored at -80 °C until analysis. Glycogen was extracted following published procedures [57, 62] and quantified using a commercial glycogen assay kit (item no. 7000480, Cayman Chemical) [57]. Briefly, cells were resuspended in 500 µL of 30% (w/v) KOH and incubated at 95 °C for 2 h in a thermomixer (Eppendorf Model 5382). Glycogen was precipitated by adding 1 mL ice-cold ethanol and incubating at -20 °C for 4 h. Precipitated glycogen was collected by centrifugation (17,000 g, 10 min, 4 °C), washed sequentially with 75% and 98% ethanol, and quantified according to the manufacturer’s protocol. Intracellular glycogen content was calculated as the mean across biological replicates and normalized to the maximum value [27], which occurred at the end of the subjective day period (20:00). Statistical significance of differences in glycogen content between subjective day and night conditions was assessed using two-tailed Student’s t-tests (*n* = 3 biological replicates). Differences with *p* < 0.05 were considered significant and *p* < 0.01 highly significant.

### Transcriptome analysis: RNA isolation, sequencing, and Data Processing

For transcriptome analysis, culture samples (10 mL) were collected at 0, 2, 4, and 8 h. Cells were harvested by centrifugation (6,000 g, 6 min, 20 °C), and pellets were flash-frozen in liquid N_2_ and stored at -80 °C prior to RNA extraction. RNA was isolated using RNeasy Plus kit and QiaShredder (QIAGEN, Hilden, Germany). Frozen cell pellets were resuspended in RLT buffer containing 1% β-mercaptoethanol, passed through a QiaShredder column, and mixed with an equal volume of 70% ethanol. Subsequent RNA extraction was performed according to the manufacturer’s protocol, followed by DNase treatment and quality assessment. RNA sequencing was performed by Azenta U.S., including rRNA depletion, additional quality assessment and Illumina sequencing (2 × 150 bp, ∼ 350 M PE reads, ∼ 105 GB, single index per lane).

Analysis of transcriptomic data was performed using R (v. 4.3.2) [67]. Raw RNA sequencing (RNA-Seq) data was processed using Rsubread [68] and mapped against the following reference genome sequences: chromosome – NC_007604.1, pANL plasmid – NC_004073.2, and pANS plasmid – NC_004990.1. The resulting RNA-Seq dataset was assessed using FastQC [69] and manually inspected to ensure quality. Raw gene counting data normalization and differential gene expression analysis were conducted using DESeq2 (v. 1.42.0) [70]. Normalized gene counts are provided in the Supplementary Dataset 1. Genes showing absolute log_2_ fold-change > 1 and adjusted *p*-value < 0.05 were considered significantly differentially expressed. The *p*-values were corrected using the Benjamini-Hochberg procedure [71]. Genes differentially expressed at subjective night conditions compared to subjective day conditions are provided in the Supplementary Dataset 2. Principal component analysis (PCA) plots were generated using ggplot2 (v. 3.5.1) [72], while heatmaps and hierarchical clustering were performed using pheatmap (v. 1.0.12). Functional enrichment analysis of differentially expressed genes was conducted using clusterProfiler (v. 4.10.1) [73]. Gene annotations were obtained from BioCyc [74], the Kyoto Encyclopedia of Genes and Genomes (KEGG) [75], Gene Ontology (GO) [76], the Clusters of Orthologous Groups (COG) [77], and the EggNOG-mapper [78].

## Results and discussion

### Circadian regulation of sucrose bioproduction

#### Design of experiment

To investigate how circadian rhythm affects photoautotrophic sucrose production in *Synechococcus elongatus* PCC 7942 cscB/SPS, we designed a time-course experiment comparing production during subjective day and night periods (Fig. 1). The strain was engineered to produce and secrete sucrose through overexpression of genes coding for sucrose phosphate synthase (*sps*) and sucrose permease (*cscB*) through induction by IPTG, as previously described [63–65]. Prior experiment under constant light the cultures were synchronized to a 14:10 light-dark cycle (light: 06:00–20:00).

The synchronized culture was divided to enable parallel sampling for the first two 8-hour periods: subjective day (12:00–20:00) and subjective night (20:00–04:00). For both conditions, samples were collected throughout the 8-hour experimental period, with time 0 corresponding to the start of each period (12:00 for subjective day and 20:00 for subjective night). Sucrose production, biomass (OD750), cell number, and glycogen content were monitored at hours 0, 0.5, 1, 2, 4, 6, and 8. For transcriptome analysis, samples were collected at hours 0, 2, 4, and 8. Both conditions were maintained under continuous light. We monitored these parameters to understand how circadian regulation orchestrates carbon allocation between growth, storage, and product synthesis.

#### Distinct growth patterns create temporal windows affecting photosynthetic production efficiency

**Fig. 2:**
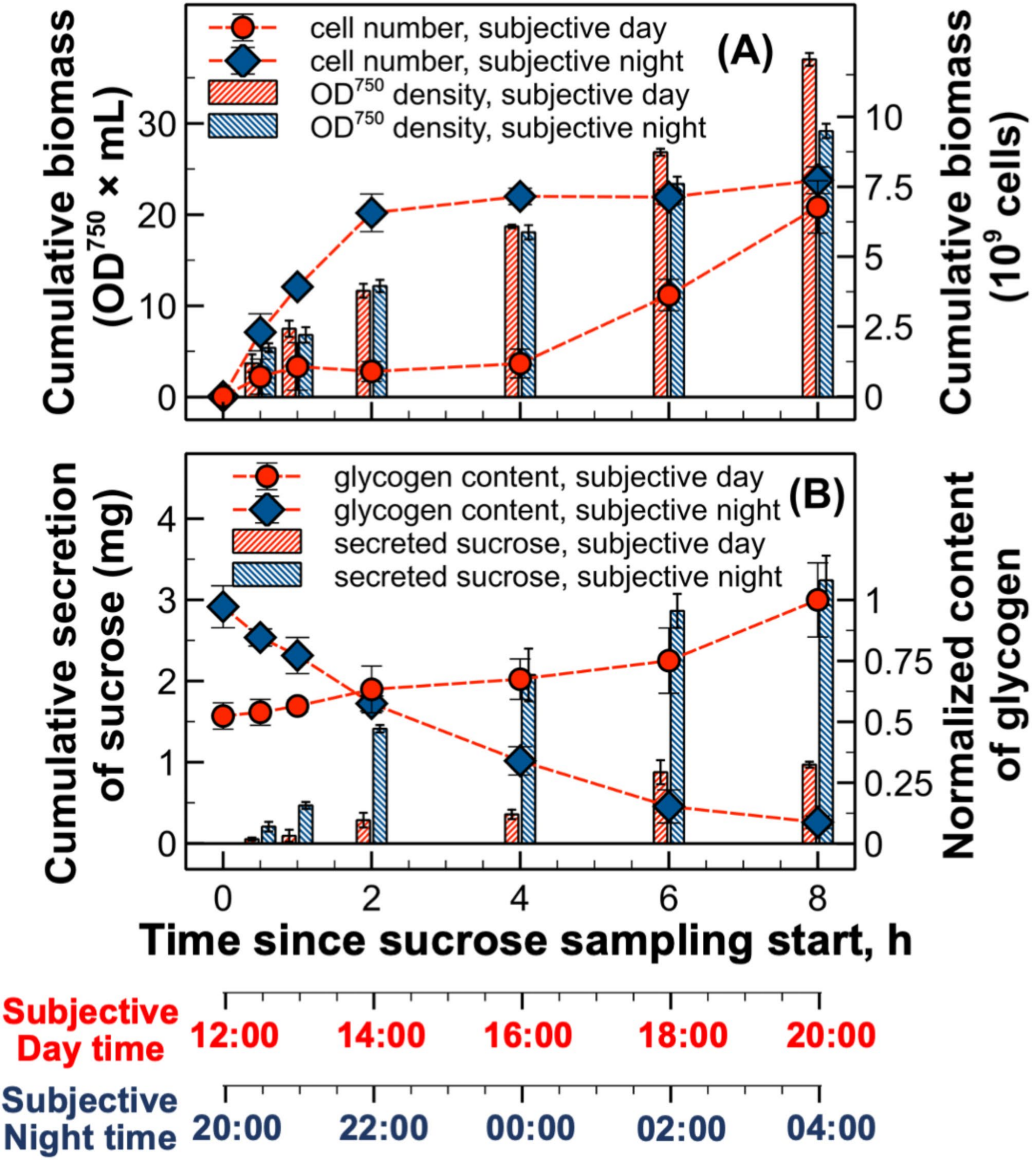
Growth characteristics and carbon allocation in *S. elongatus* PCC7942 cscB/SPS under subjective day and subjective night conditions. Data points and bars represent means of three biological replicates with error bars showing standard deviation. (**A**) Biomass accumulation measured as optical density (OD750) and cell counting (cells mL^− 1^). Cell counts showed significant differences between conditions from 1 h to 6 h (*p* < 0.01), while OD750 differences were significant at 6 h and 8 h (*p* < 0.01). (**B**) Temporal profiles of extracellular sucrose accumulation and average intracellular glycogen content normalized to the maximum value observed at the end of subjective day period. Both sucrose and glycogen levels showed significant differences between conditions throughout the experiment (*p* < 0.01), with *p* < 0.05 at 0.5 h for sucrose and at 2 h for glycogen

Growth of *S. elongatus* PCC 7942 cscB/SPS under subjective day and night conditions was monitored through two parameters: biomass accumulation (OD750) and cell number (Fig. 2A). The biomass measured by optical density (OD750) increased continuously throughout the experiment, indicating sustained carbon fixation and biomass synthesis under both conditions. During the first four hours, both cultures showed similar increases in OD750. However, the subjective day culture demonstrated higher growth rates during hours 6 and 8 of the experiment, consistent with previously observed patterns of circadian regulation of biomass accumulation [33].

In contrast to the continuous biomass accumulation, cell division patterns showed distinct temporal regulation, aligning with previous studies on circadian gating of cell division in *S. elongatus* [30, 32, 33]. Under subjective night conditions, cell division ceased during the early night hours, resulting in a plateau in cell number after two hours despite continued biomass accumulation. Conversely, under subjective day conditions, limited cell division occurred during the first 4 h (12:00–16:00), followed by significant cell proliferation during the final 4 h (16:00–20:00).

These observations confirm previous findings regarding the crucial role of the circadian clock in modulating *S. elongatus* growth patterns. Similar disconnects between biomass accumulation and cell number trajectories have been previously reported for diel-acclimated *S. elongatus* cultures (LD 12:12) after transition to constant light conditions (LL) [33]. Consistent with our findings, these studies showed continued biomass accumulation and DNA synthesis during subjective night, despite reduced or halted cell division [33]. The circadian clock acts as a major modulator of *S. elongatus* growth by controlling the transition from cell elongation to cell division initiation [30, 32, 33]. Recent studies have further demonstrated that *S. elongatus* integrates circadian signals with external cues [31, 34, 35] and other cellular processes, particularly DNA management [37–39] to regulate cell division timing.

While these cell growth and division patterns align with previously characterized circadian behaviors in *S. elongatus*, our findings reveal how these temporal growth patterns influence carbon allocation and sucrose production efficiency. The distinct growth phases create temporal windows that significantly impact the distribution of cellular resources between growth, maintenance, and product formation. When cell division is reduced during subjective night, more resources potentially become available for product synthesis, suggesting a mechanism for the observed enhancement in sucrose production during this period.

#### Carbon partitioning between synthesis of glycogen and bioproduct

The circadian state dramatically influenced both extracellular sucrose accumulation and intracellular glycogen content profiles (Fig. 2B). Under subjective day conditions, cells secreted approximately 30% of the sucrose amount produced during subjective night, despite identical light conditions. The difference in cellular glycogen dynamics was even more pronounced: cells under subjective day conditions accumulated glycogen continuously, while those under subjective night conditions showed significant glycogen depletion, even under constant light conditions.

These contrasting patterns of carbon allocation align with previous studies on circadian regulation of carbon metabolism in *S. elongatus* cscB/SPS. The rhythmic accumulation and degradation of glycogen during day and night periods, respectively, has been well-documented, with glycogen serving as a critical energy and carbon reserve during dark periods [27, 29, 79] Moreover, our observation of continued glycogen oscillations under constant light conditions is consistent with previous reports showing that diel-entrained *S. elongatus* cultures maintain circadian control over glycogen metabolism even after transitioning to constant light conditions [80].

Detailed examination of these metabolic patterns revealed complex temporal relationships between different carbon-consuming processes. The inverse relationship between glycogen accumulation and sucrose production suggests a direct competition for fixed carbon between these two pathways. During subjective day, the circadian program appears to prioritize carbon storage as glycogen, potentially limiting carbon availability for sucrose synthesis. This carbon partitioning is further complicated by the cell growth patterns observed in Sect. 3.1.2, where subjective day conditions favored cell division while subjective night conditions showed primarily cell elongation. The increased demand for carbon and energy during cell division may contribute to reduced sucrose production during subjective day. Previous studies have shown that glycogen can represent 30–60% of biomass [50–52], highlighting its significance as a major carbon sink competing with product formation.

Conversely, during subjective night, the reduction in both glycogen accumulation and cell division coincides with enhanced sucrose production, suggesting a significant shift in carbon flux distribution. The importance of this temporal orchestration is underscored by previous research showing that direct manipulation of glycogen synthesis can have unexpected effects on production. While glycogen synthesis represents a competing carbon sink, its complete elimination can lead to decreased carbon flux through heterologous pathways in engineered strains [6, 24, 53–57]. Studies have shown that blunt obstruction of glycogen synthesis may lead to detrimental distortion of cell metabolism, causing reduction in light capture and carbon fixation as well as increased sensitivity to environmental fluctuations and abiotic stress factors [58–62].

This temporal orchestration of carbon allocation between storage compounds (glycogen), cellular growth processes (elongation and division), and product synthesis (sucrose) demonstrates how circadian regulation can significantly impact the efficiency of biotechnology applications in cyanobacteria. Understanding these circadian-controlled carbon allocation patterns could provide new strategies for optimizing bioproduction in cyanobacterial systems by targeting specific temporal windows for product synthesis, rather than through direct manipulation of competing pathways.

### Molecular mechanisms underlying circadian regulation of sucrose production

#### Global transcriptome response to circadian state

**Fig. 3:**
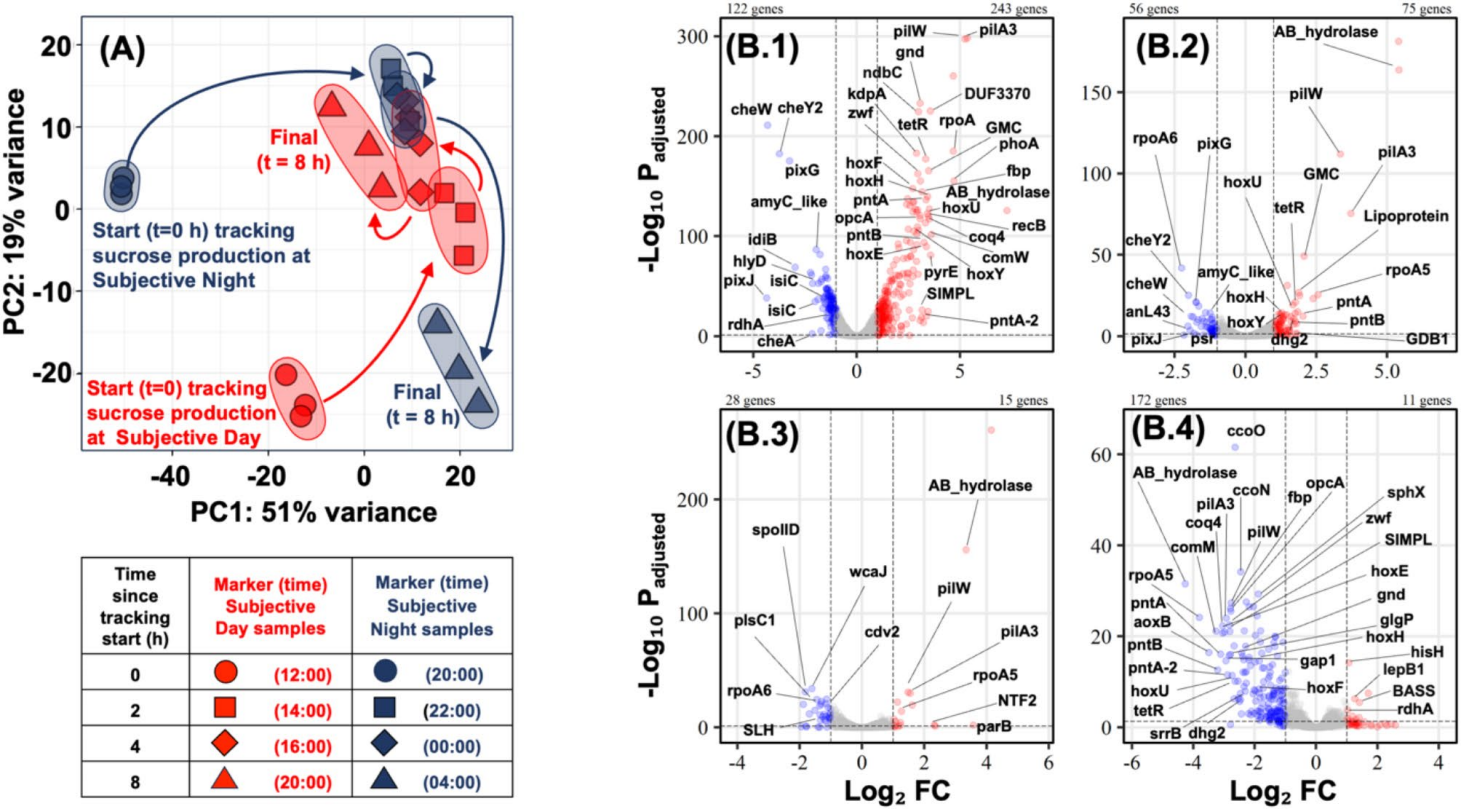
Transcriptome analysis of *S. elongatus* under subjective day and night conditions. (**A**) Principal component analysis (PCA) of global gene expression profiles. (**B**) Volcano plots showing differentially expressed genes at (**B.1**) 0 h (20:00 vs. 12:00), (**B.2**) 2 h (22:00 vs. 14:00), (**B.3**) 4 h (00:00 vs. 16:00), and (**B.4**) 8 h (04:00 vs. 20:00)

To understand the molecular basis of enhanced sucrose production during subjective night, we evaluated the impact of circadian clock on growth, division, carbon metabolism, and bioproduction in *S. elongatus* cscB/SPS through global transcriptome analysis at 0, 2, 4, and 8 h of the sucrose production experiment (Fig. 1). Of the 2,706 protein-coding genes in *S. elongatus*, 768 (28%) showed significant changes in expression across the samples. Principal component analysis (PCA) revealed distinct temporal patterns in gene expression between subjective day and night conditions (Fig. 3A). The greatest variance between subjective day and night samples was observed at 0 h (12:00 versus 20:00) across both principal components (PC1 and PC2). Sample profiles converged at 4 h (16:00 versus 00:00), showing maximum similarity, before diverging again at 8 h (20:00 versus 04:00), primarily along PC2.

Differential expression analysis revealed dynamic changes throughout the time course (Fig. 3B). The highest number of differentially expressed genes (DEGs) was observed at 0 h, with 243 up-regulated and 122 down-regulated genes under subjective night conditions (Fig. 3B.1). Functional enrichment analysis showed significant down-regulation of photosynthesis-related pathways at subjective night, including KEGG’s pathways: Photosynthesis – antenna proteins (ko00195; 14 genes, *p*adj < 10 − 3), Photosynthesis (ko00196; 11 genes, *p*adj < 10 − 3), Porphyrin metabolism (ko00860; 8 genes, *p*adj < 0.005), Carbon fixation in photosynthetic organisms (ko00710; 4 genes, *p*adj < 0.005), Ribosome (ko03010; 12 genes, *p*adj < 10 − 3), and Alanine, aspartate, glutamate metabolism (ko00250; 5 genes, *p*adj < 10 − 3).

The number of DEGs decreased to 131 at 2 h (Fig. 3B.2), with down-regulated genes enriched in Photosynthesis – antenna proteins (ko00195; 5 genes, *p*adj < 10 − 3) and Sulfur metabolism (ko00920; 5 genes, *p*adj < 10 − 3). At 4 h, the number of DEGs further decreased to 43 genes (Fig. 3B.3) with no significant functional enrichment. By 8 h (04:00 versus 20:00), the number of DEGs increased to 183 (Fig. 3B.4), with significant down-regulation of genes involved in Pentose phosphate pathway (ko00030; 7 genes, *p*adj < 0.003) and Oxidative phosphorylation (ko00190; 11 genes, *p*adj < 0.003) during subjective night. The complete list of differentially expressed genes at all time points is provided in the Supplementary Dataset 2.

These global transcriptional changes align with the physiological observations of enhanced sucrose production during subjective night, despite down-regulation of photosynthetic apparatus. The temporal patterns suggest a coordinated reprogramming of cellular metabolism that creates conditions favorable for sucrose production during subjective night, which we examine in detail in subsequent sections.

#### Differential expression of cell division and cell envelope synthesis genes

Transcriptome analysis under subjective day and night conditions (Fig. 3) supported our observations regarding circadian control of cell growth allocation between elongation and division. Several key regulatory groups showed differential expression. First, core clock components including *kaiB*, *kaiC*, *cikA*, and the master regulator *rpaA* were differentially expressed. The clock mechanisms have been extensively characterized in *S. elongatus*, with KaiA, KaiB, and KaiC forming the core oscillator complex that interacts with the master transcription factor RpaA through histidine protein kinases SasA (phosphorylates) and CikA (dephosphorylates) at different times of day [40–45].

Include the locus tag and family information in a more parallel structure: “. and transcriptional regulators (*sufR*, *idiB*, and SYNPCC7942_0599, encoding a TetR family regulator). Additionally, several response regulators were differentially expressed, including *rre1*, *srrB*, *cheY2*, *pixG*, and SYNPCC7942_2466, encoding an OmpR family regulator known to interact with SasA [81]). Previous studies have shown that the activity of numerous cell division related proteins can be adjusted by the circadian clock either directly through posttranslational modification by CikA/SasA kinases or through up-/down-regulation of expression of cell-division related genes by RpaA master regulator or RpaA-controlled sigma-factors and division regulators [40, 43, 46, 47].

**Fig. 4:**
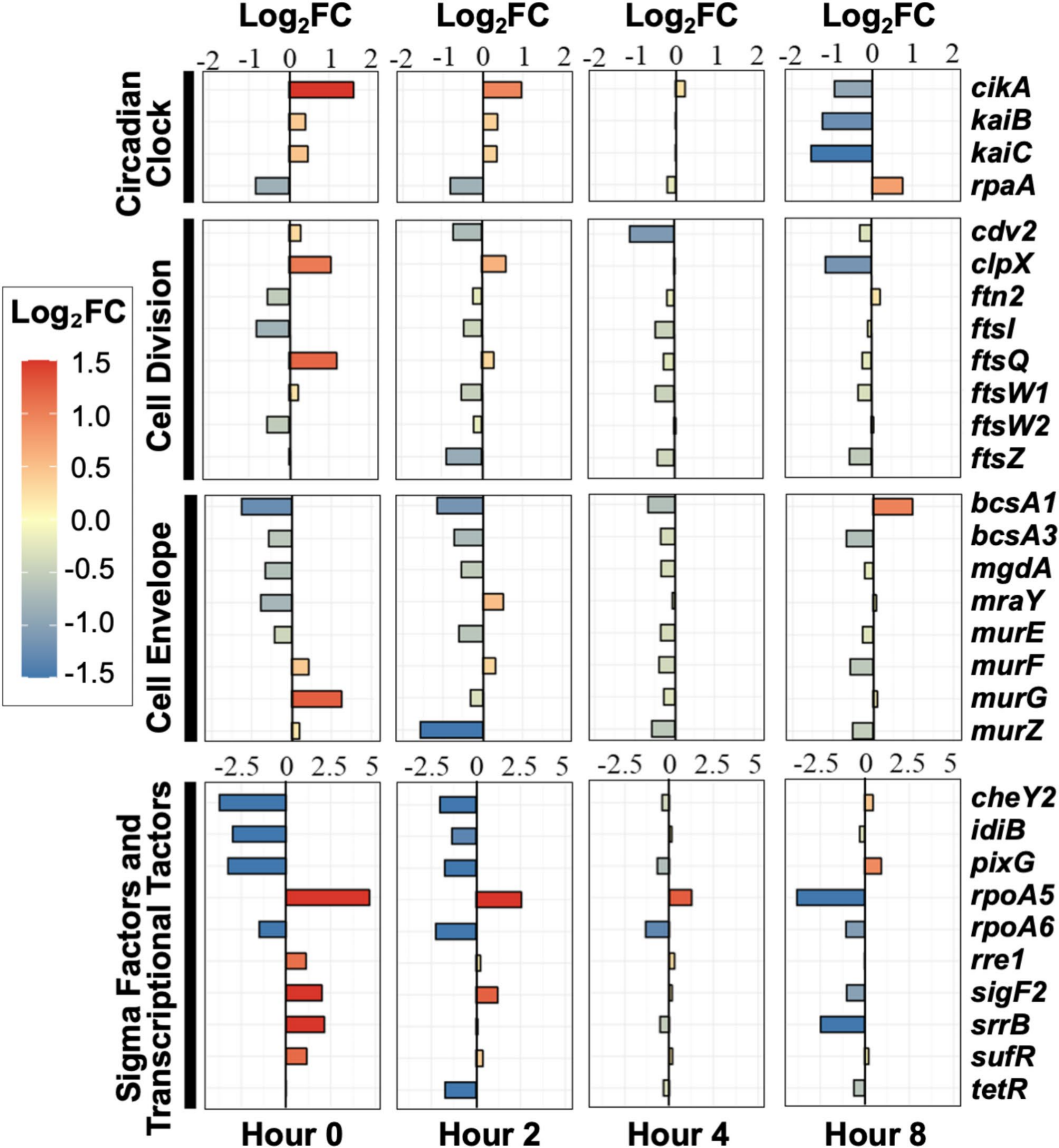
Expression profiles of circadian clock components, regulatory factors, and cell division/envelope genes. Heatmap shows differential expression between subjective night and day samples at 0 h (20:00 vs. 12:00), 2 h (22:00 vs. 14:00), 4 h (00:00 vs. 16:00), and 8 h (04:00 vs. 20:00)

Several genes directly involved in cell division showed significant expression changes (Fig. 4). These included *ftsW1* and *ftsI*, whose homologs in *E. coli* participate in peptidoglycan septa synthesis [82], and *cdv2*, a cell division gene with yet undetermined specific function [47]. The expression of key division proteins was also affected, including the ring formation protein *ftsZ* [83]), its polymerization inhibitor *sulA* which can delay cell division [84], and the RpaA-regulated chaperone *clpX*, which controls division timing through refolding or unfolding of dusk-peaking division inhibitors [85].

The circadian clock also influenced the expression of genes involved in cell envelope synthesis. These included components of the cell wall peptidoglycan (murein) biosynthesis and recycling pathways: *mraY* (known to interact with FtsZ in *Anabaena* [86]), *murE*, *murF*, *murG*, *murQ*, and *murZ*. Several *mur* genes are regulated by RpaA in *S. elongatus* and interact with cell division-related Fts proteins in *Mycobacterium tuberculosis* [40, 87]. Additionally, genes related to protective extracellular matrix assembly, such as bacterial cellulose synthesis genes *bcsA1* and *bcsA2*, showed differential expression.

The coordinated down-regulation of these division-related genes during subjective night aligns with our physiological observations of reduced cell division during peak sucrose production periods. This temporal separation of growth and production processes appears to be orchestrated at the transcriptional level through both direct clock control and downstream regulatory networks. The extensive differential expression of cell envelope-related genes supports our experimental observations that the circadian clock strongly influences the transition between cell elongation and division, creating temporal windows that favor product formation over cellular proliferation.

#### Circadian clock orchestrates energy and carbon metabolism pathways strongly impacting synthesis of target bioproduct

**Fig. 5:**
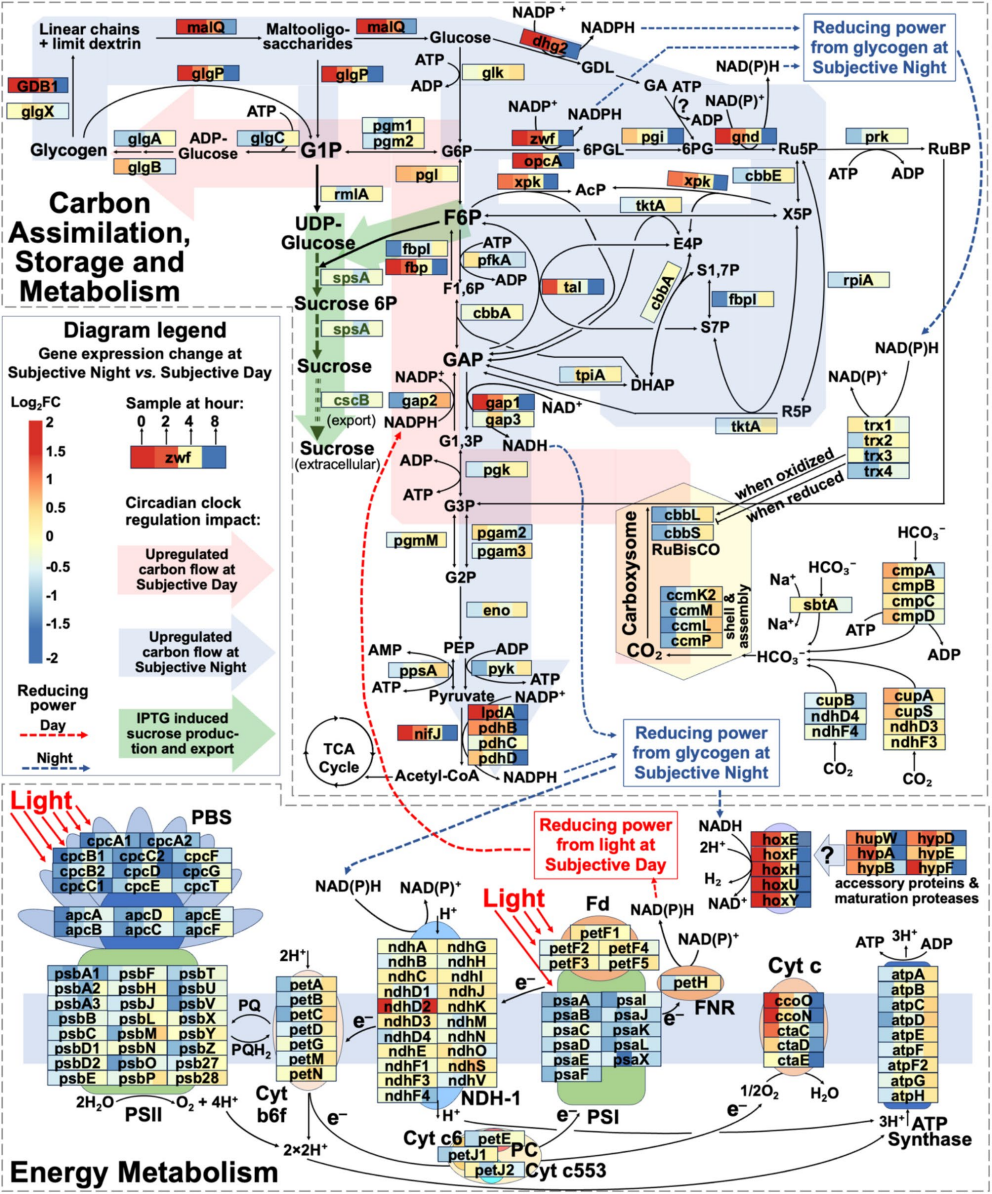
Schematic representation of energy and carbon metabolism pathways showing differential gene expression regulated by the circadian clock in *S. elongatus* PCC 7942. Colored rectangles show transcriptional responses of metabolic genes, with each rectangle containing four squares representing time points 0 h (20:00 vs. 12:00), 2 h (22:00 vs. 14:00), 4 h (00:00 vs. 16:00), and 8 h (04:00 vs. 20:00). Red boxes indicate upregulation and blue boxes indicate downregulation in subjective night relative to subjective day samples. Nodes represent major metabolites. Solid lines indicate metabolic reactions. Shaded arrows highlight major carbon flux routes: red arrows indicate pathways upregulated during subjective day (CO_2_ fixation to glycogen storage), blue arrows show pathways upregulated during subjective night (glycogen degradation through central carbon metabolism to acetyl-CoA), and green arrows represent IPTG-induced flux toward sucrose synthesis and export. Dashed lines track the generation and utilization of reducing power (NAD(P)H) during subjective night and day periods. Question marks indicate either putative protein functions or gap-filled reactions where no enzyme has been identified in the PCC 7942 genome. Brief descriptions of metabolites, genes, and proteins are provided below, while expanded descriptions of all pathway components are available in Supplementary Dataset 3

Despite constant light and CO_2_ availability throughout the experiment, the circadian clock rewired energy and carbon metabolism through dramatic modulation of gene expression profiles (Fig. 5). The temporal expression patterns, shown as colored rectangles with red (upregulated) and blue (downregulated) boxes at 0 h (20:00 vs. 12:00), 2 h (22:00 vs. 14:00), 4 h (00:00 vs. 16:00), and 8 h (04:00 vs. 20:00), revealed coordinated regulation of photosynthesis, electron transport, and carbon metabolism, providing molecular mechanisms for the enhanced sucrose production observed during the subjective night period.

##### Energy metabolism modulation under subjective night vs. day

The Energy Metabolism profile on Fig. 5 showed pronounced differential expression between subjective day and night conditions Among the most downregulated genes (Log_2_FC > 1) were light-harvesting antenna proteins, including allophycocyanin genes (*apcA-F*), linker proteins (*cpcC*, *cpcD*, and *cpcG*), and phycocyanin subunit clusters (*cpcB1A1* and *cpcB2A2*). Similar downregulation patterns were observed for components of Photosystem II (PSII) including Phycobilisome (PBS) proteins, electron transport chain (ETC) components, Photosystem I (PSI), and ATP synthase complex.

Following the electron transfer chain from photosynthesis, the strongest upregulation trend during subjective night was observed for the hydrogen-producing bidirectional hydrogenase complex genes (*hoxEFHUY*) and their associated maturation machinery *hypABDEF* and protease coded by *hupW*. These genes are organized into several operons and co-localized chromosomal regions: a small *hoxEF* operon (SYNPCC7942_0278-SYNPCC7942_0279), a *hupW-hoxHYU* operon (SYNPCC7942_2554-SYNPCC7942_2557) [88], and a cluster of hydrogenase maturation genes including *hypA* (SYNPCC7942_2553), *hypB* (SYNPCC7942_2552), and *hypF* (SYNPCC7942_2551), with *hypD* (SYNPCC7942_2386) located separately on the chromosome. This coordinated genomic organization likely facilitates the concerted expression of hydrogenase complex components and their maturation factors. The NDH-1 complex showed selective upregulation of a specific subunit, *ndhD2* (SYNPCC7942_1439), which is essential for accepting electrons from NAD(P)H into the electron transport chain. During subjective night, hydrogen production and water appear to be the terminal electron acceptors, with hox hydrogenase and cytochrome c complex playing major roles. In the absence of light, the NAD(P)H is produced through redox-active reactions of rewired carbon metabolism and utilization of glycogen reserves, as confirmed by our data.

##### Central carbon metabolism modulation under subjective night vs. day

Several Calvin-Benson-Bassham cycle genes exhibited decreased expression, indicating reduced carbon fixation activity, while genes responsible for utilizing stored carbon in the absence of light showed increased expression during dusk and early subjective night hours. This differential regulation creates distinct carbon flux patterns highlighted in Fig. 5. During subjective day, the upregulated pathways (shown by red shaded arrow) direct carbon flux from CO_2_ assimilation through carboxysomes, RuBisCO, and the CBB cycle into glycogen storage. In contrast, during subjective night, the upregulated pathways (indicated by blue shaded arrow) redirect carbon flux from glycogen through debranching/degradation to glucose, then through OPPP, glycolysis, PPP, and pyruvate metabolism to acetyl-CoA. A portion of this carbon flux is directed toward sucrose synthesis and export (depicted by green shaded arrows), which is enhanced through IPTG-induced overexpression of *spsA* (sucrose-phosphate synthase/phosphatase) and *cscB* (sucrose permease).

Glycogen degradation was enhanced through significant upregulation (log_2_FC > 2.5) of multiple enzymes: a probable glycogen debranching enzyme with glucosidase/glucanotransferase activities (putative GDB1, Synpcc7942_1574), amylomaltase (encoded by *malQ*, SYNPCC7942_1575), and glucose 1-dehydrogenase (encoded by *dhg2*, SYNPCC7942_1573). This enzymatic cascade enables efficient conversion of glycogen to gluconate through sequential formation of linear dextrins, short oligosaccharides, and glucose. While gluconate could potentially enter the oxidative pentose phosphate pathway (OPPP), no enzyme catalyzing this conversion has been identified in *S. elongatus* PCC 7942 to date. Notably, these enzymes are chromosomally co-localized, with GDB1 and *malQ* constituting a single operon. This genomic region also contains two additional upregulated genes: a dehydrogenase-like flavoprotein from the glucose-methanol-choline oxidoreductase family (Synpcc7942_1572; log_2_FC of 3.5) and a Major Intrinsic Protein (MIP) family channel protein (Synpcc7942_1571; log_2_FC of 1.7), potentially involved in glycogen catabolism or broader night metabolism.

Additionally, glycogen phosphorylase (encoded by *glgP*, SYNPCC7942_0244) represents another significant pathway for glycogen degradation, generating glucose 1-phosphate, which can either enter OPPP via conversion to glucose-6-phosphate or serve directly as a key precursor for sucrose synthesis. The GlgP-mediated route is particularly significant as it can process not only glycogen but also various degradation intermediates, suggesting that its upregulation directly contributes to enhanced sucrose production under subjective night conditions.

The oxidative pentose phosphate pathway (OPPP) demonstrated robust activation through significant upregulation of key enzymes. The *opcA*/*zwf*-encoded glucose-6-phosphate dehydrogenase and *gnd*-encoded 6-phosphogluconate dehydrogenase catalyze NADPH-generating reactions, while transaldolase (encoded by *tal*) produces fructose-6-phosphate, a critical precursor for sucrose biosynthesis. The pathway products subsequently diverge into two upregulated routes. An unexpected but significant upregulation of fructose-1,6-bisphosphatase (encoded by *fbp*) suggests a regulatory mechanism that recycles metabolic intermediates back to the OPPP, potentially preventing accumulation of sedoheptulose-7-phosphate and erythrose-4-phosphate. The second activated route, catalyzed by glyceraldehyde-3-phosphate dehydrogenase (encoded by *gap1*), directs carbon flux toward pyruvate while generating additional NADH. This metabolic configuration provides multiple advantages for sucrose bioproduction: it compensates for the absence of photosynthetic reducing power, enhances the production of the sucrose precursor fructose-6-phosphate, and maintains stable carbon flux through central metabolism.

Interestingly, among the upregulated genes during subjective night, we identified *xpk* (SYNPCC7942_2080; log_2_FC of > 1.1 at hour 0), which was recently characterized as a fructose-6-phosphate phosphoketolase. This enzyme introduces a competing reaction for fructose-6-phosphate utilization by catalyzing its cleavage to acetyl-phosphate and erythrose-4-phosphate. Additionally, Xpk can also process xylulose-5-phosphate to produce acetyl-phosphate and glyceraldehyde-3-phosphate. Despite this potentially competing pathway for the key sucrose precursor fructose-6-phosphate, the overall metabolic configuration enabled enhanced sucrose bioproduction under subjective night conditions.

##### Pyruvate metabolism and overall metabolic orchestration under subjective night vs. day

Pyruvate metabolism exhibited enhanced conversion to acetyl-CoA through two parallel pathways. The primary route showed significant differential expression of pyruvate dehydrogenase complex components, with particularly strong upregulation of lipoamide dehydrogenase (encoded by *lpdA*) and moderate upregulation of specific pyruvate dehydrogenase subunits (encoded by *pdhB* and *pdhD*). An alternative route through pyruvate: ferredoxin oxidoreductase (encoded by *nifJ*, SYNPCC7942_2384) was also activated. Notably, *nifJ* forms an operon with *hypD* (SYNPCC7942_2386), which encodes a scaffold protein essential for hydrogenase assembly, suggesting coordinated regulation of pyruvate oxidation and hydrogen metabolism. These two routes of pyruvate-to-acetyl-CoA conversion generate reducing power through different mechanisms: the pyruvate dehydrogenase complex produces NADH directly, while the NifJ-mediated pathway generates reduced ferredoxin. This dual pathway activation for pyruvate-to-acetyl-CoA suggests a sophisticated strategy for maintaining redox balance during subjective night, when photosynthetic electron transport is downregulated. The coordination of these pathways not only supports central carbon metabolism but also contributes to the overall cellular reducing power pool required for night-time metabolism.

Despite exposure to same amount of light, the orchestrated regulation of metabolic pathways under subjective night conditions appears to collectively enhance sucrose production through multiple mechanisms. The increased availability of primary sucrose precursors is achieved through coordinated upregulation of glycogen degradation enzymes and the oxidative pentose phosphate pathway. Glucose-1-phosphate is generated directly through the glycogen phosphorylase route, while fructose-6-phosphate is produced via both transaldolase reactions and fructose-1,6-bisphosphatase upregulation, and despite some diversion through competing pathways like Xpk, the net flux appears to favor sucrose synthesis. This sophisticated metabolic reconfiguration ensures efficient carbon redistribution from glycogen stores to sucrose precursors while generating substantial reducing power through multiple NAD(P)H-producing reactions, compensating for the decreased photosynthetic activity during subjective night. Such coordinated regulation demonstrates how the circadian clock orchestrates central carbon metabolism to support enhanced sucrose production while maintaining cellular redox balance.

###### Metabolites

Central carbon intermediates: G1P (glucose-1-phosphate), G6P (glucose-6-phosphate), GDL (gluconolactone), GA (gluconate), F6P (fructose-6-phosphate), FBP (fructose-1,6-bisphosphate), DHAP (dihydroxyacetone phosphate), G3P (glyceraldehyde-3-phosphate) Phosphorylated compounds: 3-PG (3-phosphoglycerate), 2-PG (2-phosphoglycerate), 1,3-BPG (1,3-bisphosphoglycerate), PEP (phosphoenolpyruvate), AcP (acetylphosphate) Pentose pathway intermediates: 6-PG (6-phosphogluconate), 6-PGL (6-phosphogluconolactone), Ru5P (ribulose-5-phosphate), RuBP (ribulose-1,5-bisphosphate), X5P (xylulose-5-phosphate), R5P (ribose-5-phosphate), E4P (erythrose-4-phosphate), S7P (sedoheptulose-7-phosphate), SBP (sedoheptulose-1,7-bisphosphate) Other metabolites: Sucrose-6P (sucrose-6-phosphate), UDP-glucose (uridine diphosphate glucose), ATP (adenosine triphosphate), ADP (adenosine diphosphate), NADPH/NADP+ (nicotinamide adenine dinucleotide phosphate reduced/oxidized forms).

###### Genes and encoded enzymes for carbon metabolism

Fixation and transport: *cbbL/S* (RuBisCO subunits), *ccmK2/L/M/P* (carboxysome proteins), *sbtA* (bicarbonate transporter), *cmpABCD* (bicarbonate transport), *cupAB* (CO_2_ hydration). CBB cycle: *prk* (phosphoribulokinase), *fbpI/fbp* (fructose-1,6-bisphosphatases). Glycolytic: *gap1/2/3* (glyceraldehyde-3-phosphate dehydrogenases), *pfkA* (phosphofructokinase), *pgmM* (phosphoglycerate mutase), *eno* (enolase). Pyruvate metabolism: *pyk* (pyruvate kinase), *ppsA* (PEP synthase), *nifJ* (pyruvate: ferredoxin oxidoreductase), *lpdA* (dihydrolipoamide dehydrogenase), *pdhBCD* (pyruvate dehydrogenase complex). Glycogen metabolism: *pgm1/2* (phosphoglucomutases), *glgC* (pyrophosphorylase), *glgA* (synthase), *glgB* (branching), *glgX* (debranching), *glgP* (phosphorylase) Glycogen degradation: *malQ* (glucanotransferase), *GDB1* (debranching), *glk* (glucokinase), *dhg2* (dehydrogenase) Sucrose metabolism: *rmlA* (thymidylyltransferase), *spsA* (phosphate synthase/phosphatase), *pgam2/3* (phosphoglycerate mutases), *cscB* (permease). Pentose phosphate pathway: *zwf*/*opcA* (glucose-6-phosphate dehydrogenase), *pgi* (phosphate isomerase), *gnd* (6-phosphogluconate dehydrogenase), *cbbE* (epimerase), *xpk* (phosphoketolase), *tktA* (transketolase), *cbbA* (aldolase), *tal* (transaldolase).

###### Genes and encoded enzymes for energy metabolism and electron transport

Photosynthetic apparatus: *apc* (allophycocyanin), *cpc* (phycocyanin), *psb* (PSII components), *psa* (PSI components). Electron transport: *pet* (cytochrome *b6f* complex and electron carriers), *cco* (cytochrome c oxidase), *cta* (cytochrome c), *cyt c553* (cytochrome *c553*). Energy coupling: *hox* (bidirectional hydrogenase), *hyp* (hydrogenase maturation), *ndh* (NDH-1 complex), *atp* (ATP synthase).

### Implications of circadian regulation for optimizing cyanobacterial bioproduction

Our transcriptome analysis, together with physiological measurements, demonstrates how circadian regulation orchestrates cellular processes in *S. elongatus*, affecting bioproduction efficiency even after induction of heterologous pathways. During subjective night, despite moderate down-regulation of photosynthetic apparatus, cultures produced approximately three-fold more sucrose compared to subjective day periods. This enhanced productivity coincided with reduced glycogen accumulation and halted cell division, indicating temporal separation of competing metabolic processes.

These findings suggest that two distinct approaches could be developed for optimizing yields in cyanobacterial bioproduction. The first approach could optimize cultivation conditions by leveraging natural circadian regulation in the bioproduction strains. Product yield could be enhanced by timing the activation of the heterologous pathway (*e.g. sps*/*cscB* in case of sucrose) to align with favorable metabolic states when competition for carbon resources is naturally reduced through decreased glycogen synthesis and halted cell division. This strategy involves coordinating production phases with the organism’s natural rhythms, implementing a “bacterial milking”-type approach [89, 90] during peak production windows. For example, activating the production pathway several hours before the night phase could maximize carbon flux toward product formation when competing processes are minimized.

The second strategy could employ synthetic biology to engineer cyanobacterial strains into controllable biocatalysts with minimized carbon waste into biomass and competing by-products. Based on results in our and other studies on circadian cycle, several regulatory targets could be modified to enhance the temporal separation of carbon storage and product formation. Key targets include regulators of glycogen metabolism (e.g., *glgP*, GDB1 pathway), cell division control elements, pathways competing for key metabolic precursors (e.g., *xpk* utilizing fructose-6-phosphate), and components of the alternative electron flow pathways for redox balancing (e.g., *ndhD2*, *hox* hydrogenase complex). Implementation of such modifications will require multiple Design-Build-Test-Learn (DBTL) cycles for optimization. However, our systematic understanding of natural temporal regulation of carbon partitioning could help reduce the number of required DBTL cycles. Key technical challenges include maintaining stable expression of heterologous pathways in large-scale cultivation, ensuring strain robustness during extended production periods, and balancing metabolic burden with productivity. These challenges can be addressed through targeted strain engineering guided by our understanding of natural regulatory mechanisms. This approach could create strains capable of efficient glycogen accumulation followed by rapid and timely conversion to desired products upon induction. The identified mechanisms of redox balance maintenance through alternative electron flow pathways could be particularly valuable in engineering robust production strains.

Development of dynamic control systems could enhance both strategies. These systems could be designed to respond to or anticipate circadian metabolic shifts through engineered promoters or metabolic sensors. For example, promoter systems could be engineered to respond to cellular redox states or glycogen levels, automatically triggering production during favorable metabolic windows. Integration of such dynamic controls with either natural circadian rhythms or engineered pathways could enable more precise temporal control over resource allocation between growth, storage, and production.

However, industrial implementation of these strategies faces several challenges that require careful consideration. First, maintaining precise temporal control over metabolic switches in large-scale cultivation systems may be technically challenging. Second, strain stability and robustness under industrial conditions, especially during extended cultivation periods, needs to be thoroughly evaluated. Additionally, the metabolic burden of heterologous protein expression could affect long-term culture viability and productivity. These challenges might be addressed through careful strain engineering for increased robustness, development of automated control systems for precise timing of induction, and optimization of cultivation conditions to minimize cellular stress. The economic viability of any implemented strategy will ultimately depend on balancing product yields with operational complexity and production costs.

The implementation of these strategies would require careful consideration of cellular energy balance and carbon partitioning. Our observation of coordinated regulation of redox balance through *ndhD2* and *hox* hydrogenase upregulation, combined with multiple routes for precursor generation through glycogen degradation and the pentose phosphate pathway, suggests that successful optimization would require integrated management of both carbon flux and reducing power. Understanding and engineering these interconnected pathways could lead to more efficient and robust production systems in cyanobacteria.

## Electronic supplementary material

Below is the link to the electronic supplementary material.

Supplementary Material 1

Supplementary Material 2

## Data Availability

Primary RNA-Seq raw measurement data are openly accessible for download at the Gene Expression Omnibus (GEO) community repository under the accession URI https://identifiers.org/geo:GSE288532. Processed transcriptomics datasets, containing normalized counts and differential gene expression results files and experimental design metadata, are openly accessible for download at the PNNL DataHub Predictive Phenomics Initiative (PPI) Project dataset catalog under 10.25584/2510500. Primary gas chromatography-mass spectrometry (GC-MS) raw measurement data are openly accessible for download at the Mass Spectrometry Interactive Virtual Environment (MassIVE) community repository under the accession URI https://identifiers.org/massive:MSV000096722 and can be formally cited under 10.25345/C59K4652C. Processed metabolomic datasets, containing targeted sucrose quantification results files and supporting metadata materials, are openly accessible for download at the PNNL DataHub Predictive Phenomics Initiative (PPI) Project dataset catalog under 10.25584/2510499. PNNL DataHub download pages include relevant computational source code information supporting data transparency and reuse where applicable.
