# Supplementary material for: Cyanobacterial circadian regulation enhances bioproduction under subjective nighttime through rewiring of carbon partitioning dynamics, redox balance orchestration, and cell cycle modulation
Source: Microb Cell Fact. 2025 Mar 8;24:56. doi: 10.1186/s12934-025-02665-5 (PMC11889915; doi:10.1186/s12934-025-02665-5)
Supplement: Supplementary file 1 — Supplementary Material 1 [file 12934_2025_2665_MOESM1_ESM.docx]

**Supplementary Information for Figure S5: Circadian Regulation of Energy and Carbon Metabolism in *S. elongatus* PCC 7942**

[A. MAJOR PATHWAY MODULES 2](#_Toc184657240)

[I. Carbon Assimilation, Storage and Metabolism 2](#_Toc184657241)

[II. Energy Metabolism 2](#_Toc184657242)

[B. METABOLITES 3](#_Toc184657243)

[1. Glycogen and Sucrose Management 3](#_Toc184657244)

[2. Calvin-Benson-Bassham Cycle 3](#_Toc184657245)

[3. Central Carbon Metabolism 3](#_Toc184657246)

[4. Energy and Electron Carriers 4](#_Toc184657247)

[C. PROTEIN COMPLEXES AND MAJOR COMPONENTS 5](#_Toc184657248)

[Energy Metabolism Components 5](#_Toc184657249)

[D. GENES AND ENZYMES 5](#_Toc184657250)

[I. Carbon Assimilation, Storage and Metabolism 5](#_Toc184657251)

[1. Carbon Uptake Systems 5](#_Toc184657252)

[2. Bicarbonate Transport 5](#_Toc184657253)

[3. Carboxysome Components and RuBisCO 6](#_Toc184657254)

[4. Calvin-Benson-Bassham Cycle 6](#_Toc184657255)

[5. Glycogen Metabolism 6](#_Toc184657256)

[6. Sucrose Metabolism 7](#_Toc184657257)

[7. Central Carbon Metabolism 7](#_Toc184657258)

[II. Energy Metabolism 8](#_Toc184657259)

[1. Photosynthetic Apparatus 8](#_Toc184657260)

[2. Electron Transport 8](#_Toc184657261)

[3. Terminal Oxidases 9](#_Toc184657262)

[4. ATP Synthesis and Hydrogenase 9](#_Toc184657263)

# A. MAJOR PATHWAY MODULES

## I. Carbon Assimilation, Storage and Metabolism

1. CO₂ fixation and carboxysome operation
2. Carbon storage through glycogen synthesis and degradation
3. Sucrose metabolism and export
4. Central carbon metabolism pathways
5. Calvin-Benson-Bassham cycle

## II. Energy Metabolism

1. Light harvesting and photosynthetic electron transport
2. NADPH generation and utilization
3. ATP synthesis
4. Hydrogen metabolism

# B. METABOLITES

### 1. Glycogen and Sucrose Management

- Linear chains + limit dextrin: Glycogen degradation products
- Maltooligosaccharides: Intermediate products of glycogen breakdown
- Glycogen: Primary carbon storage polymer
- Sucrose: Exportable carbohydrate
- Sucrose-6P: Sucrose synthesis intermediate

### 2. Calvin-Benson-Bassham Cycle

- RuBP (Ribulose-1,5-bisphosphate): CO₂ acceptor molecule
- 3PG (3-Phosphoglycerate): First stable product of CO₂ fixation
- G3P (Glyceraldehyde-3-phosphate): Key cycle intermediate
- F6P (Fructose-6-phosphate): Sugar phosphate intermediate
- S7P (Sedoheptulose-7-phosphate): Seven-carbon intermediate
- R5P (Ribose-5-phosphate): Pentose phosphate
- Ru5P (Ribulose-5-phosphate): RuBP precursor
- X5P (Xylulose-5-phosphate): Pentose phosphate pathway intermediate
- E4P (Erythrose-4-phosphate): Four-carbon intermediate

### 3. Central Carbon Metabolism

- G1P (Glucose-1-phosphate): Glycogen metabolism intermediate
- G6P (Glucose-6-phosphate): Central metabolic hub
- GDL (Gluconolactone): Spontaneous hydrolysis product
- GA (Gluconate): Spontaneous hydrolysis product of GDL
- 6PGL (6-Phosphogluconolactone): Oxidative PPP intermediate
- 6PG (6-Phosphogluconate): Oxidative PPP intermediate
- PEP (Phosphoenolpyruvate): High-energy intermediate
- 2PG (2-Phosphoglycerate): Glycolytic intermediate
- Pyruvate: Central metabolic intermediate
- Acetyl-CoA: Gateway to TCA cycle
- AcP (Acetyl phosphate): High-energy compound

### 4. Energy and Electron Carriers

- ATP/ADP: Energy currency
- NADPH/NADP⁺: Primary photosynthetic reducing equivalent
- NADH/NAD⁺: Respiratory reducing equivalent
- PQ/PQH₂: Plastoquinone/Plastoquinol
- Fd: Ferredoxin
- PC: Plastocyanin

# C. PROTEIN COMPLEXES AND MAJOR COMPONENTS

## Energy Metabolism Components

- PSII: Photosystem II complex
- PBS: Phycobilisome
- PSI: Photosystem I complex
- Cyt b6f: Cytochrome *b*6*f* complex
- PC: Plastocyanin
- Cyt c553: Cytochrome c553
- Cyt c6: Cytochrome c6
- Fd: Ferredoxin
- FNR: Ferredoxin-NADP oxidoreductase

# D. GENES AND ENZYMES

## I. Carbon Assimilation, Storage and Metabolism

### 1. Carbon Uptake Systems

- **High-Affinity CO₂ Uptake System 1**
  - *cupA*: CO₂ hydration protein A
  - *cupS*: Stabilizing protein for CupA
  - *ndhD3*: NDH-1 complex specialized subunit
  - *ndhF3*: NDH-1 complex specialized subunit
- **High-Affinity CO₂ Uptake System 2**
  - *cupB*: CO₂ hydration protein B
  - *ndhD4*: NDH-1 complex specialized subunit
  - *ndhF4*: NDH-1 complex specialized subunit

### 2. Bicarbonate Transport

- **High-Affinity Bicarbonate Transport System**
  - *cmpA*: Bicarbonate-binding protein
  - *cmpB*: Transmembrane permease
  - *cmpC*: ATP-binding protein
  - *cmpD*: ATP-binding protein
- **Sodium-Dependent Bicarbonate Transport**
  - *sbtA*: Sodium-dependent bicarbonate transporter

### 3. Carboxysome Components and RuBisCO

- *ccmK2*: Major shell protein
- *ccmL*: Vertex protein
- *ccmM*: Internal scaffold protein
- *ccmP*: Shell protein
- *cbbL*: RuBisCO large subunit
- *cbbS*: RuBisCO small subunit

### 4. Calvin-Benson-Bassham Cycle

- *prk*: Phosphoribulokinase
- *fbpI*: Fructose-1,6-bisphosphatase I
- *fbp*: Fructose-1,6-bisphosphatase II
- *gap1*: NAD⁺-dependent glyceraldehyde-3-phosphate dehydrogenase
- *gap2*: NADP⁺-dependent glyceraldehyde-3-phosphate dehydrogenase
- *gap3*: Type I glyceraldehyde-3-phosphate dehydrogenase
- *tktA*: Transketolase
- *tal*: Transaldolase

### 5. Glycogen Metabolism

- *pgm1*: Phosphoglucomutase 1
- *pgm2*: Phosphoglucomutase 2
- *glgC*: ADP-glucose pyrophosphorylase
- *glgA*: Glycogen synthase
- *glgB*: 1,4-alpha-glucan branching enzyme
- *glgX*: Isoamylase-type debranching enzyme
- *GDB1*: Glycogen debranching enzyme
- *malQ*: 4-alpha-glucanotransferase
- *glgP*: Glycogen phosphorylase

### 6. Sucrose Metabolism

- *spsA*: Sucrose-phosphate synthase/phosphatase
- *cscB*: Sucrose permease (heterologous from *E. coli*)
- *rmlA*: Glucose-1-phosphate thymidylyltransferase

### 7. Central Carbon Metabolism

#### Glycolysis/Gluconeogenesis

- - *glk*: Glucokinase
  - *pgi*: Glucose-6-phosphate isomerase
  - *pfkA*: 6-phosphofructokinase
  - *pgam2*: Phosphoglycerate mutase 2
  - *pgam3*: Phosphoglycerate mutase 3
  - *pgmM*: Phosphoglycerate mutase
  - *eno*: Enolase
  - *pyk*: Pyruvate kinase
  - *ppsA*: Phosphoenolpyruvate synthase

#### Pyruvate:Ferredoxin Oxidoreductase Pathway

- - *nifJ*: Pyruvate:ferredoxin oxidoreductase

#### Pyruvate Dehydrogenase Complex

- - *lpdA*: Dihydrolipoamide dehydrogenase
  - *pdhB*: Pyruvate dehydrogenase E1
  - *pdhC*: Dihydrolipoamide acetyltransferase
  - *pdhD*: Dihydrolipoamide dehydrogenase

## II. Energy Metabolism

### 1. Photosynthetic Apparatus

#### Phycobilisome Components

- - *cpcA1*/*cpcA2*: Phycocyanin α-subunits
  - *cpcB1*/*cpcB2*: Phycocyanin β-subunits
  - *cpcC1*/*cpcC2*: Rod linker proteins
  - *cpcD*: Small rod linker
  - *cpcG*: Rod-core linker
  - *cpcE*: Phycocyanobilin lyase E
  - *cpcF*: Phycocyanobilin lyase F
  - *cpcT*: Bilin lyase

#### Photosystem II Components

- - *psbA1*/*psbA2*/*psbA3*: D1 proteins
  - *psbB*: CP47 protein
  - *psbC*: CP43 protein
  - *psbD1*/*psbD2*: D2 proteins
  - *psbEF*: Cytochrome b559
  - Additional *psb* genes (U-Z): Various structural components

#### Photosystem I Components

- - *psaA*-*psaB*: Core proteins
  - *psaC*: FA/FB iron-sulfur centers
  - *psaD*-*psaL*: Various functional components

### 2. Electron Transport

#### Cytochrome Complex

- - *petA*-*petN*: Cytochrome b6f components
  - *petE*: Plastocyanin
  - *petF1*-*petF5*: Ferredoxins
  - *petH*: Ferredoxin-NADP⁺ reductase
  - *petJ1*/*petJ2*: Cytochrome c553

#### NDH-1 Complex

- - *ndhA*-*ndhN*: Core complex components
  - *ndhD1*/*ndhD2*: Specialized subunits
  - *ndhF1*/*ndhF2*: Specialized subunits

### 3. Terminal Oxidases

- *ccoO*: Cytochrome c oxidase subunit II
- *ccoN*: Cytochrome c oxidase subunit I
- *ctaC*: Cytochrome c oxidase subunit II
- *ctaD*: Cytochrome c oxidase subunit I
- *ctaE*: Cytochrome c oxidase subunit III

### 4. ATP Synthesis and Hydrogenase

#### ATP Synthase

- - *atpA*-*atpH*: F₁F₀ ATP synthase components

#### Bidirectional Hox [NiFe]-Hydrogenase Complex

- - *hoxE*: Diaphorase subunit
  - *hoxF*: NADH-binding subunit
  - *hoxU*: Iron-sulfur protein
  - *hoxY*: Small subunit
  - *hoxH*: Large subunit

#### Bidirectional Hox [NiFe]-Hydrogenase Assembly proteins

- - *hypA*: Nickel insertion
  - *hypB*: GTPase
  - *hypD*: Iron center assembly
  - *hypE*: Carbamoyl dehydratase
  - *hypF*: Carbamoyl phosphate processing
  - *hupW*: Hydrogenase-specific protease
